# Prophylaxis or Prevention of Dental Decay

**Published:** 1870-04

**Authors:** Andrew F. M’Lain

**Affiliations:** Professor of Institutes of Dentistry in the New Orleans Dental College


### PROPHYLAXIS OR PREVENTION OF DENTAL DECAY.

BY ANDREW F. M'LAIN, D. D. S., M. D.,

Professor of Institutes of Dentistry in the New Orleans Dental College.

Read before the Southern Dental Association.

MR. PRESIDENT AND GENTLEMEN:

From the great amount of industry, zeal and intelligence which have been, and are still being directed, through journalistic and other sources, by gentlemen of attainments and skill, on the various branches of the *healing art*, to the treatment and cure of diseases pertaining to their respective specialties, it appears somewhat singular, that with the varying, nay, sometimes doubtful success which attends and has always attended such efforts, and from the prevalency and constant recurrence of those same diseases, so little attention has been paid to *prophylaxis*, or what is more familiarly known as *prevention*.

If this observation is true of the medical art proper, how much more applicable is it to Dental surgery than to any other of the allied branches of medicine, inasmuch as the causes of Dental decay being rather of a physico-vital character, they are better understood, and are, therefore, more easily guarded against.

As prolific as Dental literature has become, and numerous and able, taken as a class, as are the practitioners of this benificent art, very few among them, indeed, have thought of applying that homely old adage, that "an ounce of prevention is worth a pound of cure," but it would seem that the *sine qua non* of their aim and ambition consisted in combating diseases already established, instead of warding them off.

Admitting that Dentists, as well as physicians, are not usually consulted until the patient has become ill, or is seriously threatened with illness, or the ravages of caries have already begun in the Dental organs, still much good might be done, in default of that elementary knowledge which should be possessed, at least, by all heads of families, if not generally, how to preserve the teeth from those disorders to which they are so commonly subject, by Dental practitioners adopting the plan of imparting to their patients during their professional intercourse with them, or to the parents or guardians, when these are too immature in age to profit by such instructions, some practical information concerning the nature and chemical composition of the teeth; the general causes of their decay; how they may be acted upon by certain agents taken in the mouth as food or drink; the necessity of certain kinds of food being used in order that the integrity of their organic structure may be maintained; by explaining to them the relation which the teeth bear to other portions of the organism, and how by reactions from other organs (the stomach for instance) the teeth may become affected; by giving them an idea of the office of the teeth and at the same time setting forth how much the quality of the blood, and consequently, the proper nutrition of those organs depends upon the perfect mastication of the food; and above all, they should be fully impressed with the necessity of absolute cleanliness of the teeth, together with the observance of such other hygienic and preventive measures as may be deemed appropriate to the case.

The French, as a general thing, are much better versed in those rules having reference to diet and ordinary hygiene than the American people, for children are taught at an early age, to exercise some discrimination in the choice of food, by selecting such articles as are healthful and nutritious, and to eschew those that are naturally indigestible, or whose qualities are impaired, or whose effects are otherwise pernicious. And what is of great moment, they are made to observe some regularity in the periods for taking their food, besides restricting the quantity thus taken within the proper bounds, while, at the same time, avoiding it at improper hours. They are likewise indoctrinated as soon as sufficient intelligence is manifested, with some general ideas of sanitary laws, which, however crude they may be, often prove of infinite benefit to them, and to those who, perchance, may come under their guidance.

Though such teachings may not possess more than the faintest glimmering of science, yet by their practical utility, children are insensibly led to understand how to avoid all unnecessary exposures and those grosser modes of life so inimical to health. I would not be understood as implying that the Dentist should become the *mentor* of his patients, and thus usurp the sphere of the natural guardians, but he may, whilst engaged in the legitimate performance of his professional duties, do much toward correcting popular errors and pernicious habits that are no doubt destroying millions of teeth annually.

The hygienic management of the teeth needs no formal introduction, for not unfrequently the subject is brought up by patients themselves, who manifest their solicitude by asking, "Doctor, how can I prevent my teeth from decaying?" At other times, the perceptible neglect, or the evident abuse of the Dental organs, will suggest to the operator the propriety of advising, after however, ascertaining from the patient something of his or her habits ; in short has discovered in what the error consists, some rational course of conduct, by which, if the evil can not be remedied, at least, that no further mischief may ensue. If neglect of cleanliness has given rise to the trouble, it becomes the Dentist's duty, to explain, that the teeth being of an alkaline nature, the accumulation of food about them, especially of vegetable matters, a fermentative process, accelerated, no doubt, by the warmth and moisture of the mouth is established, and which results in an acid product, capable of acting on the enamel of the teeth, but more energetically on the bony structure ; thereby causing that decomposition called Dental decay. On the other hand, should the well being of the teeth be jeopardized by their abuse, whether from hard usage or from the habitual use of acid ingesta or drinks, such habits should be unqualifiedly condemned, by showing how the biting of hard substances is liable to crack or fracture the enamel and thus expose the dentine to the operation of external agents ; and that the frequent contact of acids, having as they do, a natural chemical affinity for the alkaline earths of the teeth, induces a disintegration which eventually terminates in the usual phenomena following upon Dental caries.

It should likewise be shown, that the physical or constitutional integrity of the teeth depends very materially upon the healthy functions of the stomach, for the habitually faulty performance of the digestive process causes the dissolution of the teeth through acid reactions, as well as inducing, as has already been intimated, a deterioration in their organic structure from not receiving sufficient nutriment from a circulating medium, rendered through dyspeptic tendencies, too poor in amount and quality of its nutrient elements.

In regard to the influence which diet exerts on the textures, it should be pointed out to patients, that certain kinds of food produce certain effects on the structures, hence, the great necessity for a judicious choice in the articles to be used. Although it is generally admitted that man thrives best on a mixed diet, that is composed of animal and vegetable substances, yet it is equally certain, that a due proportion of certain principles should exist in that food whether mixed or not, in order that each part of the organism may derive the elements of which it is composed. Hence, the different tissues require different kinds of nourishment, inasmuch as waste is going on continually, each organ, therefore, deteriorating if not constantly supplied with pabulum suited to its nature. Some textures demand albumen for their sustenance, some saccharine or amylaceous properties, others oleaginous or phosphoric or sulphurous, while again, others derive nourishment from lime in different forms, magnesia, selica, etc., all entering into various combinations to suit the requirement of individual organs and of the whole assemblage of organs and each having the power to select the food possessing the properties peculiar to its organization. The phosphate of lime with a trace of the flouride or silicate of calcium together with the carbonate of lime and magnesia, enter largely into the composition of bones, but still more abundantly in that of the teeth. Now, it is perfectly evident, that in consequence of the intermolecular waste or deterioration already noticed as being constantly in operation; the various portions of the organism can be maintained in their normal standard, only by being well supplied with those materials so essential to their constitutional nature, a condition naturally and very readily effected if such matters be present, or allowed to remain in the food.

The various species of grain such as wheat, rye, corn and many others, possess naturally those earthy or calcareous principles alluded to in sufficient quantity for the demands of the economy, but *man*, from a vain desire to improve on nature, has foolishly devised, through the process known as "bolting," the means of divesting the grain of the greater portion of its calcareous substances which always exist on its exterior, thus depriving the bread that is commonly eaten of its most essential properties.

It has been frequently observed, that the Scotch, English, German and French peasantry, and all those nations whose diet consists of the plainest kind of fare, a large proportion of which being coarse brown bread, are endowed as a general rule, with a much better quality of Dental structures than those of the more luxurious classes of society.

Unfortunately until the majority of the people in this age of refinement and false living are educated to a better standard with regard to culinary knowledge, it can not be expected that they will go back to primitive or natural habits and customs in the preparation of their food, especially, here in the United States, where the most unscientific, I might say, execrable cookery is done, but Dentists being conscious of a gradually increasing defect in the structural condition of the teeth of the younger generations, whether produced by the removal of the necessary earthy elements of their composition from the food, or from their original absence or deficiency in such aliment, might, whenever the demand for them seems to exist, recommend their regular medicinal administration, as a component part of each meal, in such quantity and form as the case may require.

Of course, in adults, where the teeth are naturally deficient in the earthy constituents, such organs will be less amenable to remedial measures, and are, therefore, incapable of any marked constitutional improvement, but I have long held it quite possible, that by the early administration, (commencing in *utero*) of the phosphate preparations of lime, to be able not only to avert this condition of the teeth, but to impart a good structural quality to every living offspring.

It, therefore, follows, if this hypothesis be true, that the most favorable time for the accomplishment of this object, would be to begin with the mother during gestation, previous to the formative stage of the teeth in the child, and continue until the second dentition shall have been completed, a period in life when the absorption and appropriation of all the constituents that go to make up the body are well known to be most active. There are cases too, later in life, when their exhibition would not be unappropriate, but it will be readily apprehended, that in them, it would be more for the purpose of maintaining the teeth at a given standard in quality.

Now, as regards the more eligible *forms* in which the lime salts should be given, I am disposed to accord the preference to the hypo-phosphate or the lacto-phosphate as being more easily assimilated. At all events, this hypothesis received considerable support from a series of experiments recently instituted by Messrs. Dusart and Blanke, of Paris, with the lacto-phosphate on guinea pigs. These experiments consisted in taking a number of these animals of the same age and size, and feeding them all alike, but giving to one half of them which had been separated from the others, a certain quantity of the lacto-phosphate of lime in addition to the usual daily food. To mark still more closely the physiological effects of the drug in health and disease, some of each class had a limb broken. Some length of time was allowed to elapse, and then all the pigs were killed and their bones respectively weighed and noted. It was found after repeated trials, that the bones of those to which the lime had been given averaged thirty-three per cent more in weight than the bones of those from which the lime had been withheld.

It may be objected, that in view of the natural differences existing between the higher and the lower animals some differences in its physiological effects on the human species might obtain, but the effects of medicines, generally, on the two orders when they do occur, are usually so nearly similar, and are so slight as to be merely nominal.

In conclusion, gentlemen, I will reaffirm, that, imparting to patients the various sanitary rules respecting the hygienic management of the teeth, is a duty which Dental advisers owe to them as well as to themselves. There need be no fear, as some have expressed, that the vocation of the Dentist would be destroyed by thus instructing the people—that there would be no more decay of these organs, but it should be remembered, that so long as the artificial state into which mankind has been drawn continues, and as long as accidents and the various other ills to which flesh is heir obtain, just so long will the teeth be liable to disorders, and, therefore, require Dental aid.

Practitioners of the art of Dental surgery whose professional education is so defective as to render them incapable of giving suitable advice for the proper management of the teeth, should retire at once from the field of practice, and leave it to those who are able to advise as well as to execute. I trust, however, that the day is not far distant, when every member within the fold of the profession, will not only be competent to inform patients how to preserve their teeth, but also to convey the knowledge how to impart good Denta organs to unborn generations.

